# Diagnostic Challenges in a Young Man with a Suspected Mast Cell Disorder, Dysplastic Bone Marrow Morphology, and a *ZRSR2* Mutation

**DOI:** 10.3390/hematolrep17060064

**Published:** 2025-11-25

**Authors:** Riccardo Dondolin, Nawar Maher, Annalisa Andorno, Sayed Masoud Sayedi, Mohammad Reshad Nawabi, Andrea Patriarca, Gianluca Gaidano, Riccardo Moia

**Affiliations:** 1 Division of Hematology, Department of Translational Medicine, Università del Piemonte Orientale, 28100 Novara, Italy; nawar.maher@uniupo.it (N.M.); sayedmasoud.sayedi@uniupo.it (S.M.S.); 20041622@studenti.uniupo.it (M.R.N.); andrea.patriarca@uniupo.it (A.P.); gianluca.gaidano@med.uniupo.it (G.G.); riccardo.moia@uniupo.it (R.M.); 2 Azienda Ospedaliero-Universitaria Maggiore della Carità di Novara, 28100 Novara, Italy; annalisa.andorno@maggioreosp.novara.it; 3 Azienda Ospedaliero-Universitaria di Alessandria, 15121 Alessandria, Italy; 4 Division of Pathology, Department of Health Sciences, Università del Piemonte Orientale, 28100 Novara, Italy

**Keywords:** *ZRSR2*, mast cell disorders, myelodysplastic syndromes, CHIP

## Abstract

**Background and Clinical Significance:** Mastocytosis and mast cell activation syndrome (MCAS) include conditions in which patients manifest signs, symptoms, and laboratory findings consistent with mast cell activation and can only be diagnosed in the presence of specific criteria. Mutations of *ZRSR2*, a gene involved in RNA splicing, are not closely associated with mast cell disorders, but rather with myelodysplastic syndromes development. **Case Presentation:** We report a case of a 37-year-old man who was referred to our institution for anaphylaxis after a bee sting and elevated serum tryptase levels (17.8 ng/mL in the first sample and 19.2 ng/mL in the second sample). Complete blood count was unremarkable. Bone marrow biopsy showed signs of dysplasia and some CD25+ mast cells. ASO-qPCR and targeted myeloid NGS analysis did not detect the *KIT* p.D816V mutation, but rather showed the presence of a pathogenetic variant of the *ZRSR2* gene (p.S447_R448del) with a variant allele frequency of 7.4%. Mastocytosis could not be diagnosed based on the established diagnostic criteria. The patient’s symptoms were not recurrent and tryptase release was not event-related; therefore, a diagnosis of MCAS could not be made either. Taken together, these findings led to the diagnosis of clonal hematopoiesis of indeterminate potential (CHIP). A watch and wait strategy consisting of clinical evaluations, blood tests, and cardiovascular risk assessment was initiated. **Conclusions:** This case report highlights the importance of combining clinical and laboratory findings, hematopathology, and molecular analyses to establish the most probable diagnosis in challenging cases. It also underscores the possible relevance of identifying predisposing conditions, such as CHIP, in order to guide counseling and follow-up strategy.

## 1. Introduction

Mastocytosis is a condition characterized by proliferation and tissue infiltration of clonal mast cells, which accumulate in one or multiple organs. The disease is associated with a huge variety of clinical manifestations [1]. The two main subtypes of mastocytosis, namely cutaneous mastocytosis (typical of children) and systemic mastocytosis (SM), can manifest with or without skin involvement. SM is categorized into five variants: indolent systemic mastocytosis (ISM), smoldering systemic mastocytosis (SSM), systemic mastocytosis with an associated hematologic neoplasm (SM-AHN), aggressive systemic mastocytosis (ASM), and mast cell leukemia (MCL) [2,3]. The diagnosis of mastocytosis is confirmed if patients manifest one major criterion and one minor criterion or meet three minor criteria [2,3,4]. Conversely, mast cell activation syndrome (MCAS) includes a number of conditions in which patients have signs and symptoms consistent with mast cell activation, have evidence of systemic mast cell-mediator release, but do not harbor mastocytosis criteria [1,5]. Symptoms of mast cell activation must be severe, systemic, and recurrent. An event-related mast cell-mediator release and regression of symptoms with anti-mediator therapy are required [6]. The cases of MCAS where an underlying mastocytosis is found are termed clonal MCAS or primary MCAS [5,7].

The oncogenic *KIT* p.D816V mutation is detectable in >80% of all patients with mastocytosis. Other somatic gene alterations implicated in some cases of advanced systemic mastocytosis include mutations in *TET2*, *SRSF2*, *ASXL1*, *RUNX1*, *CBL*, *JAK2, NRAS*, and *KRAS* [4]. In contrast, mutations of *ZRSR2*, a gene involved in RNA splicing, are not closely associated with mast cell disorders, but rather with myelodysplastic syndromes (MDS) development [8,9].

## 2. Case Report

We report a case of a 37-year-old Caucasian man with an unremarkable clinical history who was referred to our institution for anaphylaxis after a bee sting. During the event, the patient experienced cutaneous erythema, hypotension, and tachycardia. The patient had already been seen by an allergy specialist, who trained the patient in the use of an adrenaline autoinjector and suggested hematological evaluation to exclude mastocytosis. When evaluated at our institution, the patient was asymptomatic and physical examination was negative, in particular for cutaneous lesions, rash, and dermographism. Blood tests were unremarkable except for elevated levels of serum tryptase in two different blood samples: 17.8 ng/mL in the first sample (performed 16 days after anaphylaxis) and 19.2 ng/mL in the second sample, collected one month after the first one (upper limit of normal: 15 ng/mL) (Table 1). Serum tryptase was elevated, including in a third sample collected three months after the second one. Abdominal ultrasound was negative for hepatomegaly and splenomegaly.

**Figure 1:**
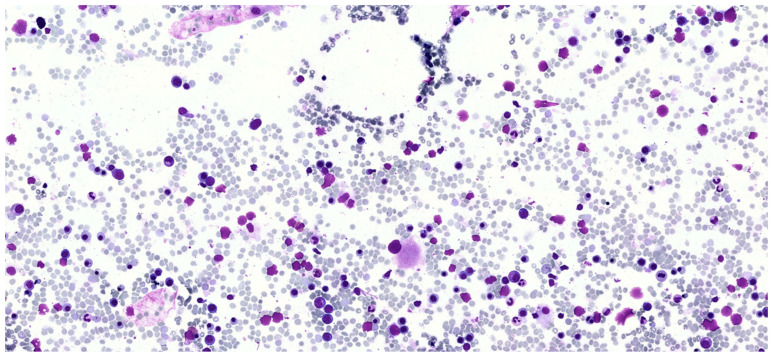
Bone marrow smear for cytomorphological examination. The image shows dysplastic features observed in the patient’s bone marrow smear, including a dysplastic megakaryocyte and cytoplasmic as well as internuclear bridges in the erythroid lineage.

**Figure 2:**
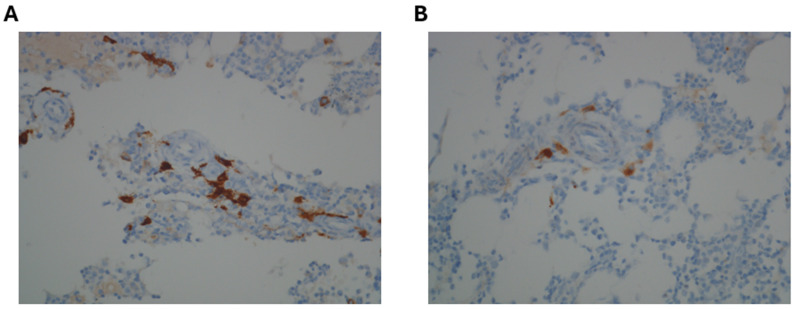
Immunohistochemical staining performed on bone marrow biopsy. (**A**) Immunohistochemical staining for CD117: CD117 positivity, combined with morphological evaluation, allows for the effective identification of mast cells. (**B**) Immunohistochemical staining for CD25: the figure shows the partial positivity for CD25 in some spindle-shaped mast cells, which suggests the presence of a mast cell disorder.

Considering that serum tryptase elevation and allergic reactions to Hymenoptera stings are a relatively common manifestation in mast cell disorders, a bone marrow aspiration and biopsy were performed. Morphological examination of the bone marrow blood smear revealed the presence of moderate trilinear dysplasia, such as micromegakaryocytes and megakaryocytes with hypolobated nuclei, cytoplasmic hypogranulation in granulocytic lineage, and cytoplasmic and internuclear bridges in the erythroid lineage (Figure 1). The *KIT* p.D816V mutation assessed by ASO-qPCR was negative and the bone marrow karyotype was normal. *PDGFRA*, *PDGFRB*, *FGFR1*, and *PCM1*::*JAK2* gene fusions were also tested using specific FISH probes and were negative. Targeted myeloid next-generation sequencing (NGS) analysis showed the presence of a pathogenetic variant of the *ZRSR2* gene (p.S447_R448del) with a variant allele frequency of 7.4%. Interestingly, NGS analysis did not detect any mutations in all the other *KIT* exons (2, 8–11, 13, 17, 18) analyzed nor in other genes previously reported in patients with advanced systemic mastocytosis (*TET2*, *SRSF2*, *ASXL1*, *RUNX1*, *CBL*, *JAK2*, *NRAS*, and *KRAS*). Bone marrow histology did not show the presence of aggregates of more than 15 mast cells per high-power field, but several spindle-shaped CD117-positive mast cells with partial expression of CD25 were detected (Figure 2A,B). Spindle-shaped mast cells were <25% of the total mast cells. It was not possible to assess CD30 expression by immunohistochemistry nor to perform rare-event flow cytometry to detect aberrant mast cells. Not having performed these tests represents a limitation, as it prevented a more detailed characterization of bone marrow mast cell population. Bone marrow cellularity was within normal limits (30–40%), and signs of dysplasia were confirmed in >10% of megakaryocytic, granulocytic, and erythroid lineages. The percentage of myeloid blasts in the bone marrow was <1%.

According to mastocytosis diagnostic criteria [1,2,3,4,5] (Table 2), the patient had one/two minor criteria for the disease, but no major criteria. More precisely, spindle-shaped mastocytes were present, but they were <25% of the total mast cells and, therefore, the first minor criterion was not respected. The second minor criterion was not respected either since the *KIT* p.D816V mutation was negative. The third minor criterion was respected since the patient’s mast cells expressed CD25. The fourth minor criterion was borderline since serum tryptase levels were persistently elevated and very close to 20 ng/mL, though not >20 ng/mL as required by the diagnostic criteria. On these grounds, the diagnosis of clonal mast cell disorder could not be established and *ZRSR2* mutation was attributed to clonal hematopoiesis of indeterminate potential (CHIP) [1,2,3,4,5]. Given the detection of CHIP, a watch and wait strategy with a clinical evaluation and blood tests every six months to monitor development of cytopenias was initiated, together with periodic cardiovascular risk assessment. Two years after initial presentation, the complete blood count and differential are unremarkable, and the patient is asymptomatic.

## 3. Discussion

With mastocytosis excluded, the three main differential diagnoses considered in this patient were MCAS, a myelodysplastic syndrome, or CHIP. Another diagnostic hypothesis could have been hereditary alpha tryptasemia (HαT), which can be associated with elevated serum tryptase levels. However, in the absence of a positive family history and other suggestive symptoms (gastrointestinal, respiratory, musculoskeletal, or neurological), this hypothesis was ruled out and genetic testing of *TPSAB1* gene was not performed. The clinical presentation, serum tryptase elevation, and the presence of spindle-shaped CD25+ mast cells suggested the presence of a MCAS, but the patient did not have recurrent symptoms [5,6,7]. Tryptase levels measurement delayed from the anaphylactic reaction, rather than during or immediately after the event, represents a limitation in the diagnostic process, potentially compromising the accuracy of the differential diagnosis. Indeed, confirmation of a diagnosis of MCAS requires evidence of an event-related rise in tryptase levels (Table 3). Therefore, it will be crucial to obtain the patient’s immediate tryptase measurements in case of future anaphylactic episodes. Moreover, although serum tryptase levels were consistently elevated in the patient, they did not reach the threshold for one of the minor criteria of mastocytosis [2,3]. In this respect, it is also important to consider that serum tryptase levels are not specific to mastocytosis but can also be elevated in up to 25% of MDS [10]. Moreover, dysplastic bone marrow features were observed during both the bone marrow smear observation and the histological analysis of the bone marrow biopsy. In addition, mutations of *ZRSR2* are not reported to be implicated in mastocytosis, whereas they occur in other myeloid neoplasms, such as MDS [8,9]. Interestingly, *ZRSR2* mutations in patients with a diagnosis of MDS are associated with male sex and with an indolent clinical phenotype, and our patient closely reflected this description [11]. However, in the absence of peripheral cytopenias, the diagnosis of MDS could not be made and the *ZRSR2* mutation was ascribed to clonal CHIP [12]. Therefore, although the initial clinical presentation of the patient was suggestive of mastocytosis/MCAS, and some bone marrow findings were reminiscent of MDS, these hypotheses were not confirmed by the integration of molecular, morphological, and biochemical findings. Instead, the integration of all findings led to the diagnosis of CHIP, with incidental dysplastic bone marrow features.

**Figure 3:**
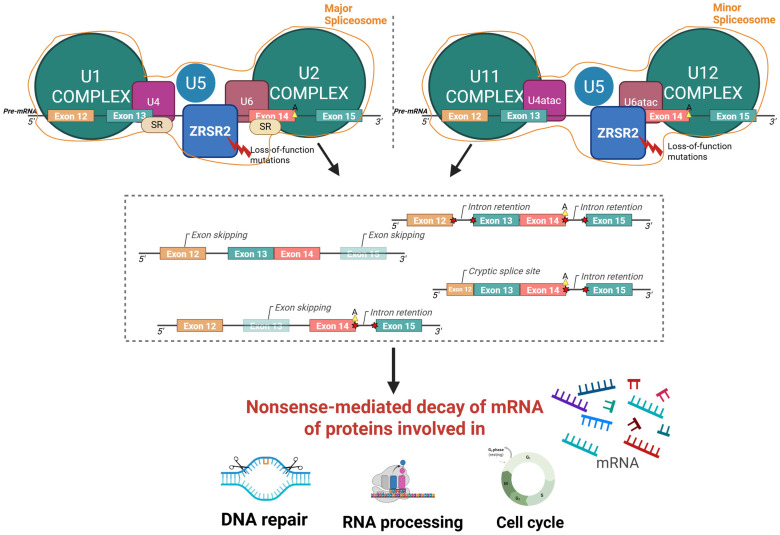
Graphical representation of the function and biological consequences of the *ZRSR2* mutation. Loss-of-function mutations in *ZRSR2* gene impair the spliceosome complex, leading to abnormal splicing events such as exon skipping, intron retention, and cryptic splice sites formation. These aberrant splicing patterns trigger nonsense-mediated decay of mRNA, ultimately altering the production of proteins essential for DNA repair, RNA processing, and cell cycle regulation. Major spliceosome complex: assembly of U1/U2/U4/U5/U6 small nuclear ribonucleoproteins (snRNPs); minor spliceosome complex: assembly of U11/U12/U4atac/U6atac snRNPs; SR: serine/arginine-rich proteins; A: branchpoint adenosine; 5′/3′: RNA strand orientation; red bolt: loss-of-function mutations in *ZRSR2*; red stars: aberrant splicing events. Created using BioRender; Gaidano, G (2025) https://BioRender.com/p5j4m85 (accessed on 3 November 2025).

*ZRSR2*, located on the X chromosome (Xp22.1), encodes a component of the RNA spliceosome complex (Figure 3) [13]. Splice gene mutations, mainly involving *SF3B1*, *SRSF2*, *ZRSR2*, and *U2AF35*, are among the most common molecular alterations detected in MDS and also in patients harboring CHIP [13,14]. In contrast to mutations in other genes involved in the splicing process, which are typically clustered in a limited number of hotspot regions, mutations in *ZRSR2* have been found to be more broadly distributed throughout the gene [15]. Alterations affecting the arginine-serine-rich domain, especially the R446 amino acid residue, have been reported in the context of MDS, suggesting the relevance of this gene region in MDS development [15]. Interestingly, previous studies demonstrated that *ZRSR2* loss of function, which is typical of MDS and other myeloid neoplasms, can impair inflammation and immune response pathways (i.e., Toll-like receptors signaling), providing a possible explanation for the dysregulated reaction to a bee sting that was observed in this patient, regardless of the specific hematologic diagnosis [8,16].

The rarity and the overlapping definitions of mast cell disorders make this case a diagnostic challenge, with a substantial risk of under- or overdiagnosis [5,7]. This case highlighted that the most common symptoms and laboratory findings of mastocytosis (i.e., anaphylaxis after a bee sting and elevated tryptase levels) do not always lead to mastocytosis/MCAS diagnosis, further underscoring the need for bone marrow evaluation in suspected cases. The current diagnosis of CHIP harboring a *ZRSR2* pathogenetic mutation can lead to two different clinical scenarios: (*i*) the association between CHIP and the occasional finding of dysplastic bone marrow may represent an early detection of a subsequent MDS, or (*ii*) the typical clinical presentation of mastocytosis/MCAS and the finding of CD25+ spindle-shaped mast cells in the bone marrow may represent an early detection of a subsequent overt mastocytosis/MCAS predisposed by a CHIP *ZRSR2*-mutated clone.

## 4. Conclusions

This case report highlights the synergistic role in hematology of combining hematopathology with molecular analyses to establish the most probable diagnosis in challenging cases, especially when the clinical presentation is subtle or ambiguous. It also underscores the possible importance of identifying potential predisposing conditions, such as CHIP, in order to guide follow-up strategies in patients considered at risk of developing overt hematologic diseases.

Clinical monitoring for potential new episodes of anaphylaxis, along with regular assessment of complete blood count and serum tryptase levels, will be continued over time to further clarify this challenging scenario. In the event of an anaphylactic episode, tryptase levels will also be measured during and shortly after the acute phase. If clinically indicated (i.e., presence of suggestive symptoms), genetic testing will be considered to rule out HαT. If new cytopenias emerge, bone marrow re-evaluation will be performed, also including rare-event flow cytometry for aberrant mast cell detection and CD30 immunohistochemical staining. The size of the *ZRSR2*-mutated myeloid clone will also be closely monitored.

## Figures and Tables

**Table 1:** Patient’s blood tests.

First Sample		Second Sample	
WBC	6320/μL	WBC	5670/μL
ANC	3120/μL	ANC	2190/μL
Hemoglobin	15.6 g/dL	Hemoglobin	14.9 g/dL
Platelets	329.000/μL	Platelets	288.000/μL
LDH	352 U/L (ULN 450 U/L)	LDH	310 U/L (ULN 450 U/L)
Tryptase	17.8 ng/mL (ULN 15 ng/mL)	Tryptase	19.2 ng/mL (ULN 15 ng/mL)

WBC: white blood cells; ANC: absolute neutrophil count; LDH: lactate dehydrogenases; ULN: upper limit of normal.

**Table 2:** Diagnostic criteria of systemic mastocytosis.

**The diagnosis of systemic mastocytosis can be made when the major criterion and at least 1 minor criterion are present, or when ≥3 minor criteria are present.**
**Major criterion:**
Multifocal dense infiltrates of mast cells (≥15 mast cells in aggregates) detected in sections of bone marrow and/or other extracutaneous organ(s).
**Minor criteria:**
In biopsy sections of bone marrow or other extracutaneous organs, >25% of the mast cells in the infiltrate are spindle-shaped or have atypical morphology or >25% of all mast cells in bone marrow aspirate smears are immature or atypical. Detection of an activating point mutation at codon 816 of *KIT* in the bone marrow, blood, or another extracutaneous organ. Mast cells in bone marrow, blood, or another extracutaneous organ express CD25, CD2, and/or CD30, in addition to normal mast cell markers. Serum total tryptase is persistently >20 ng/mL, unless there is an associated myeloid neoplasm, in which case this parameter is not valid.

**Table 3:** Diagnostic criteria of mast cell activation syndrome.

Episodic symptoms consistent with mast cell-mediator release affecting two or more organ systems Skin: urticaria, angioedema, flushing Gastrointestinal: nausea, vomiting, diarrhea, abdominal cramping Cardiovascular: hypotensive syncope or near syncope, tachycardia Respiratory: wheezing Naso-ocular: conjunctival injection, pruritus, nasal stuffiness
2 A decrease in the frequency or severity or resolution of symptoms with anti-mediator therapy
3 Evidence of an elevation in a validated urinary or serum marker of mast cell activation and documentation of elevation of the marker above the patient’s baseline during a symptomatic period on at least two occasions; if baseline tryptase levels are persistently >15 ng/mL, documentation of elevation of the tryptase above baseline on one occasion
4 Primary (clonal) and secondary disorders of mast cell activation ruled out

## Data Availability

The original contributions presented in this study are included in the article. Further inquiries can be directed to the corresponding author.
